# Risk factors for functional decline among survivors of Gram-negative bloodstream infection: A prospective cohort study

**DOI:** 10.1371/journal.pone.0259707

**Published:** 2021-11-17

**Authors:** Adi Turjeman, Fidi Koppel, Erica Franceschini, Dafna Yahav, Giovanni Dolci, Tanya Babich, Roni Bitterman, Ami Neuberger, Nesrin Ghanem-Zoubi, Antonella Santoro, Noa Eliakim-Raz, Barak Pertzov, Anat Stern, Yaakov Dickstein, Elias Maroun, Hiba Zayyad, Marianna Meschiari, Jihad Bishara, Elad Goldberg, Claudia Venturelli, Cristina Mussini, Mical Paul, Leonard Leibovici

**Affiliations:** 1 Department of Medicine E, Rabin Medical Center, Beilinson Hospital, Petah-Tikva, Israel; 2 Sackler Faculty of Medicine, Tel Aviv University, Tel Aviv, Israel; 3 Infectious Diseases Institute, Rambam Health Care Campus, Haifa, Israel; 4 Clinic of Infectious Diseases, University of Modena and Reggio Emilia, Modena, Italy; 5 Infectious Diseases Unit, Rabin Medical Center, Beilinson Hospital, Petah-Tikva, Israel; 6 The Ruth and Bruce Rappaport Faculty of Medicine, Technion–Israel Institute of Technology, Haifa, Israel; 7 Pulmonary Division, Rabin Medical Center, Beilinson Hospital, Petah-Tikva, Israel; 8 Department of Medicine F, Rabin Medical Center, Beilinson Hospital, Petah-Tikva, Israel; 9 Clinical Microbiology Lab, University of Modena and Reggio Emilia, Modena, Italy; IRCCS Policlinico S.Donato, ITALY

## Abstract

**Objective:**

To identify risk factors for functional decline after hospitalization for Gram-negative bacteremia.

**Patients and methods:**

A prospective cohort study based on a randomized controlled trial conducted between January 1, 2013 and August 31, 2017 in Israel and Italy. Hospitalized patients with Gram-negative bacteremia who survived until day 90 and were not bedridden at baseline were included. The primary end point was functional decline at 90 days.

**Results:**

Five hundred and nine patients were included. The median age of the cohort was 71 years (interquartile range [IQR], 60–80 years), 46.4% (236/509) were male and 352 of 509 (69%) patients were independent at baseline. Functional decline at 90 days occurred in 24.4% of patients (124/509). In multivariable analysis; older age (odds ratio [OR], 1.03; for an one-year increment, 95% confidence interval [CI] 1.01–1.05), functional dependence in instrumental activities of daily living at baseline (OR, 4.64; 95% CI 2.5–8.6), low Norton score (OR, 0.87; 95% CI 0.79–0.96) and underlying comorbidities: cancer (OR, 2.01; 95% CI 1.14–3.55) and chronic pulmonary disease (OR, 2.23 95% CI 1.12–4.42) and longer length of hospital stay (OR 1.09; for one-day increment, 95% CI 1.04–1.15) were associated with functional decline. Appropriate empirical antibiotic treatment was associated with lower rates of functional decline within 90 days (OR, 0.4; 95% CI 0.21–0.78).

**Conclusions:**

Patients surviving bloodstream infections have poor long term trajectories after clinical recovery and hospital discharge. This has vast implications for patients, their family members and health policy makers.

## Introduction

Gram-negative bacteremia is a major cause of morbidity and mortality in both hospitalized and community-dwelling patients. Bacteremia is the seventh ranked leading cause of death in the United States and Europe with in-hospital mortality rates of up to 30% [1–3]. The worldwide incidence of Gram-negative bacteremia has increased over the last decades. While the occurrence of bacteremia increased, mortality rates remained unchanged or in some cases decreased slightly [4–6]. Consequently, the number of bacteremia survivors is rising steadily [7]. The main focus of current management guidelines is interventions dealing with the reduction of short term mortality. Data regarding the long-term trajectory of sepsis and bacteremia among survivors are limited.

Functional decline among bloodstream infection survivors has major implications for patients, families, and the health care system. Following hospitalization due to sepsis, patients develop 1 to 2 new limitations of daily living activities which can negatively affect quality of life [8]. For the elderly, functional decline is the leading post discharge complication [9].

The present study was conducted on patients with Gram-negative bacteremia who participated in a randomized controlled trial (RCT) and survived until day 90 [10]. We aimed to determine risk factors for functional decline at 90 days among survivors of Gram-negative bacteremia and to evaluate the rate of functional decline in these patients.

## Methods

### Study design and patients

The design of the RCT has been described in detail previously [10]. Briefly, this was a randomized, multicenter, open-label, non-inferiority trial conducted between 2013 and 2017 in two academic centers in Israel (Rabin Medical Center, Beilinson Hospital; Rambam Health Care Campus) and one academic center in Italy (Hospital of Modena). Six hundred and four inpatients with Gram-negative bacteremia that were afebrile and hemodynamically stable for at least 48 hours, were randomized.

Bacteremia was defined as growth of Gram-negative bacteria in one or more blood cultures. Patients with bacteremia from the following sources were eligible for inclusion: urinary tract, intra-abdominal, respiratory tract, central venous catheter, or skin and soft tissue infection or an unknown source. Exclusion criteria were: other sources of infection requiring prolonged treatment, fever or hemodynamic instability in the 48 hours prior to randomization, uncontrolled focus of infection, polymicrobial growth involving Gram-positive bacteria, specific pathogens (Brucella, Salmonella), or immunosuppression (human immunodeficiency virus, neutropenia, or recent allogeneic stem cell transplantation). The RCT’s design as an investigator initiated trial, particularly the broad inclusion criteria and the limited exclusion criteria, were meant to represent our target population.

Eligible patients were identified based on a daily review of microbiology laboratory reports of Gram-negative bacteremia. Patients fulfilling inclusion criteria were then approached by an infectious diseases physician from the study team. Patients that provided written informed consent were randomized in a 1:1 ratio to receive 7 days (intervention) or 14 days (control) antibiotic treatment. Functional capacity at baseline and hospital admission was documented for all participants in the RCT. Discharge, thirty and ninety day functional capacity were pre-planned secondary outcomes.

For the present observational prospective cohort study, we excluded: (i) Patients who did not survive until day 90 (N = 68) and (ii) patients who were bedridden before the acute infection (N = 27). The final analysis included 509 patients. The RCT and the observational study were approved by the local ethics board of each participating center: Rabin Medical Center Institutional Review Board (Israel), Rambam Institutional Review Board (Israel) and Comitato Etico dell’Area Vasta Emilia Nord (Italy). All RCT’s participants provided written informed consent before their inclusion.

### Definitions and outcomes

The following data were obtained from the patient, family, attending physician and computerized medical records: baseline patient characteristics, demographics, cognitive status, comorbidities, presentation of infection, infection characteristics, antibiotic treatment, microbiological data, clinical management of infection and outcomes. Appropriate empirical treatment was defined as covering antibiotic therapy that matched the in vitro susceptibility of the Gram-negative pathogen in blood administered within 48 hours. Hospital acquired infections were defined as those occurring after 48 hours of hospital admission. Diarrhea was defined as ≥3 episodes per day for at least 2 days. Readmission was defined as any hospitalization occurring after discharge until 90 days.

Functional assessment was assessed at 5 time points: baseline, hospital admission, discharge from hospital, 30 days and 90 days; and coded into a 4-point scale: independent; functional dependence in instrumental activities of daily living (I-ADL): preparing meals, managing money, shopping, doing housework, using a telephone, leaving the house; functional dependence in basic ADL (B-ADL): eating, dressing, toileting, bathing, transferring; bedridden.

Data regarding functional status at baseline, hospital admission and discharge were collected in real time during hospital stay. Post discharge data related to functional capacity at 30 and 90 days after infection onset were collected via telephone interviews. Information was provided by the patient, the primary caretaker or the family physician.

The primary endpoint was functional decline, defined as a decline of 1-point or more from baseline.

### Statistical analysis

Data were expressed as frequencies (percentages) for categorical variables, mean ± standard deviation for normally distributed continuous variables and as median and interquartile range (IQR, 25–75 percentiles) for non-normally distributed continuous variables.

Univariate analysis was conducted for all independent variables. Characteristics of subjects who had functional decline were compared to those who did not using the t-test or Mann–Whitney U-test (as appropriate based on their distribution) for continuous variables. The Chi-square test was used for categorical variables. Variables that were statistically significantly associated with functional decline (2-sided *P value* ≤ .05) were included in a multivariable analysis according to their clinical relevance. Multicollinearity was tested to examine the correlations between independent variables.

We used a logistic regression model to perform a multivariable analysis (Generalized Linear Models procedure of SPSS) and identify variables independently associated with 90-day functional decline. Four models were examined in order to adjust the best fit using the Akaike Information Criterion (AIC). Results are presented as odds ratios (OR) with 95% confidence intervals (CI). Statistical analyses were performed using IBM SPSS Statistics, version 25.

## Results

### Functional capacity of 604 bacteremia patients included in the RCT at 5 time-points

**Fig 1:**
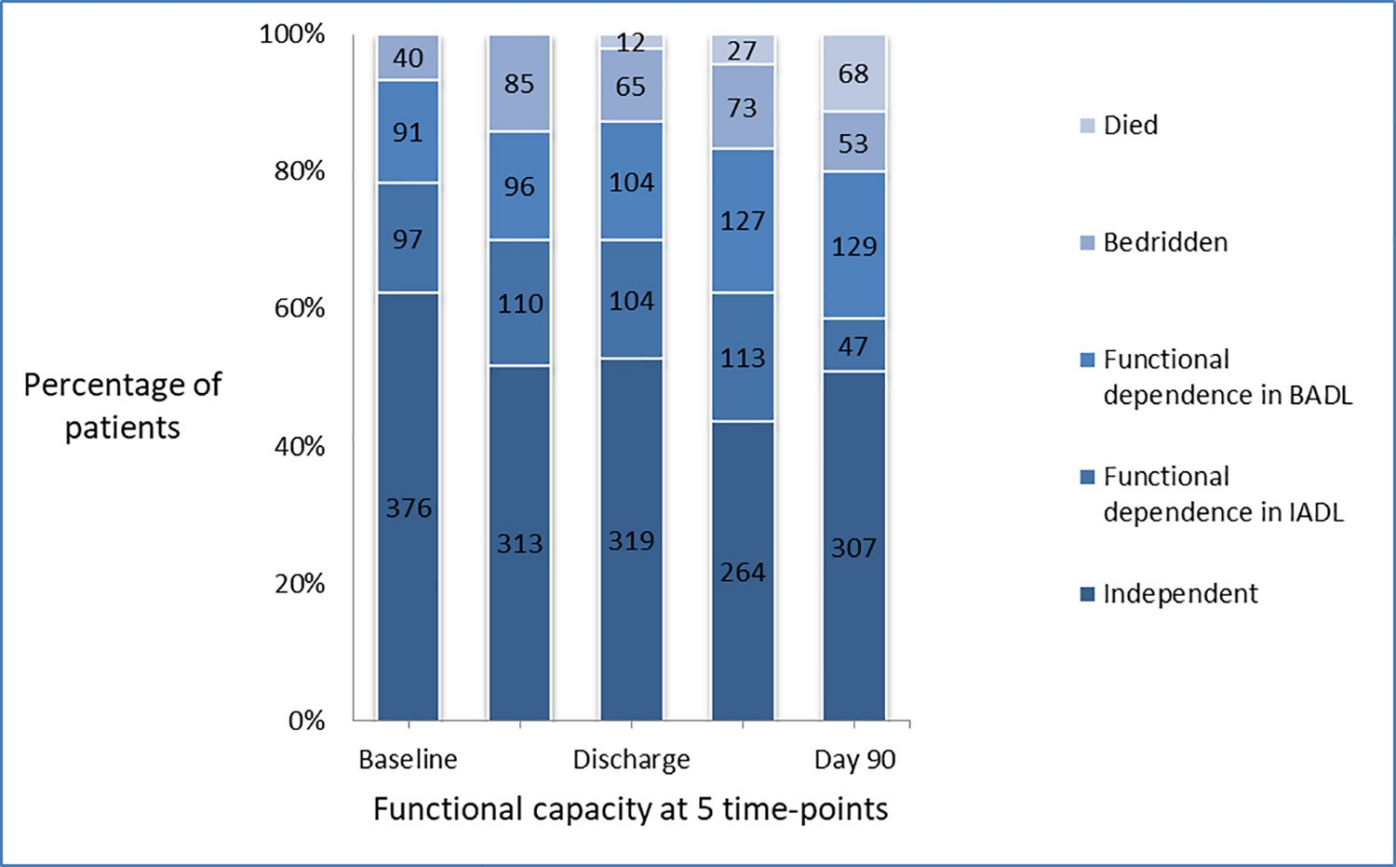
Description of functional capacity by a 4-point scale of 604 bacteremia patients at 5 time-points.

Functional capacity on a 4-point scale at baseline, hospital admission, hospital discharge, 30 and 90 day follow up is shown in Fig 1. At baseline, 376 patients were functionally independent, 97 patients were dependent for instrumental activities of daily living (IADL), 91 patients were dependent for basic activities of daily living (BADL) and 40 patients were bedridden (62.3%, 16.1%, 15.1%, 6.6%, respectively). At 90 days, 18% (69/376) of patients who were independent at baseline became non-independent to some degree. The rate of independent patients decreased from 62.3% (376/604) at baseline to 50.8% (307/604) at 90 days. Moreover, the rate of patients dependent for basic ADL has increased from 15.1% at baseline to 21.3%, 90 days after bacteremia onset (91/604 versus 129/604, respectively). The inconsistency in numbers of bedridden patients during the study period is explained by the number of deaths. The all-cause mortality rate within 90 days was 11.3% (68/604) (Fig 1).

### Functional capacity of 536 bacteremia survivors at baseline and at day 90

The interaction between functional capacity of 536 bacteremia survivors at 2 time points: baseline and day 90, is presented in Table 1. One fifth of bacteremia survivors experienced functional decline (124/536) during study period, among them, almost 40% (46/124) had a significant functional decline of 2 or more levels. No change in functional capacity was observed among 388 patients. Functional capacity improved in 24 patients. Among 536 survivors, 27 patients (5%) were bedridden at baseline, and therefore not included in the analysis (Table 1).

**Table 1:** Descriptive statistics of patients’ functional capacity at baseline and at day 90^a^^,^^b^.

Functional capacity at baseline	Functional capacity at day 90			
	Independent (N = 307)	Functional dependence in IADL (N = 47)	Functional dependence in BADL (N = 129)	Bedridden (N = 53)	Total (N = 536)
**Independent**	290 (94.5)	24 (51.1)	28 (21.7)	10 (18.9)	352 (65.7)
**Functional dependence in IADL**	14 (4.6)	20 (42.6)	41 (31.8)	8 (15.1)	83 (15.5)
**Functional dependence in BADL**	2 (0.7)	3 (6.4)	56 (43.4)	13 (24.5)	74 (13.8)
**Bedridden**	1 (0.3)	0 (0)	4 (3.1)	22 (41.5)	27 (5)

^a^ Data are presented as number (%).

^b^ Abbreviations: IADL, instrumental activities of daily living; BADL, basic activities of daily living.

### Characteristics of 509 patients included in the univariate analysis

Baseline characteristics of 509 inpatients with Gram-negative bacteremia that were not bedridden at baseline and survived to day 90 are presented in Tables 2 and 3. Patients’ median age was 71 years (interquartile range [IQR], 60–80 years), 46.4% (236/509) were male, and 73.5% lived with their families (374/509). While almost 70% of patients (352/509) were independent at baseline, most of the cohort was non-employed (80.6%, 400/509). The main source of bacteremia was the urinary tract (352/509 [69%]), and about one third were hospital-acquired (142/509).

**Table 2:** Patient characteristics^a^^,^^b^.

Variable	No functional decline at 90 days, N = 385	Functional decline at 90 days, N = 124	Entire cohort N = 509	P value
Age, median (IQR), years ^c^	69 (58.5–79)	76.5 (64.3–83)	71 (60–80)	< .001
Sex, male	173 (44.9)	63 (50.8)	236 (46.4)	.25
Disturbed consciousness	15 (3.9)	10 (8.1)	25 (4.9)	.06
Marital status				.48
Single	23 (6)	3 (2.4)	26 (5.1)	
Married	244 (63.4)	80 (64.5)	324 (63.7)	
Divorced	38 (9.9)	11 (8.9)	49 (9.6)	
Widowed	79 (20.5)	29 (23.4)	108 (21.2)	
Number of children, median (IQR)	2 (1–3.5)	3 (1–4)	2 (1–4)	.3
Functional capacity at baseline				< .001
Independent	290 (75.3)	62 (50)	352 (69.2)	
Functional dependence in instrumental activities of daily living (IADL)	34 (8.8)	49 (39.5)	83 (16.3)	
Functional dependence in basic activities of daily living (BADL)	61 (15.8)	13 (10.5)	74 (14.5)	
Residency				.36
Home with family	282 (73.2)	92 (74.2)	374 (73.5)	
Home alone	66 (17.1)	15 (12.1)	81 (15.9)	
Nursing home	15 (3.9)	8 (6.5)	23 (4.5)	
Other	22 (5.7)	9 (7.3)	31 (6.1)	
No employment	286 (76.5)	114 (93.4)	400 (80.6)	< .001
**Comorbidities**				
Charlson comorbidity score, median (IQR) ^d^	2 (0–3)	2 (1–4)	2 (1–3)	< .001
Congestive heart failure	21 (5.5)	18 (14.5)	39 (7.7)	.001
Cerebrovascular disease	40 (10.4)	23 (18.5)	63 (12.4)	.02
Diabetes				.02
No diabetes	251 (65.2)	67 (54)	318 (62.5)	
Diabetes without end-organ damage	104 (27)	38 (30.6)	142 (27.9)	
Diabetes with end-organ damage	30 (7.8)	19 (15.3)	49 (9.6)	
Chronic pulmonary disease	37 (9.6)	26 (21)	63 (12.4)	.001
Malignancy	79 (20.5)	36 (29)	115 (22.6)	.05
Norton score, median (IQR)	19 (16–20)	16 (14–18)	18 (15–19)	< .001

^a^ Data are presented as number (%) unless otherwise indicated.

^b^ Abbreviations: IQR, interquartile range.

^c^ Age-per one year increment

^d^ S3 Table [24].

**Table 3:** Infection characteristics and management ^a^^,^^b^.

Variable	No functional decline at 90 days, N = 385	Functional decline at 90 days, N = 124	Entire cohort N = 509	P value
Predisposition				
Hospital-acquired infection	94 (24.4)	48 (38.7)	142 (27.9)	.002
Central venous catheter prior to infection	28 (7.3)	7 (5.6)	35 (6.9)	.54
Peripheral catheter prior to infection	73(19)	43 (34.7)	116 (22.8)	< .001
Infection characteristics and presentation				
Bacteria type ^c^				.11
*Escherichia coli*	260 (67.5)	72 (58.1)	332 (65.2)	
*Klebsiella* spp	51 (13.2)	16 (12.9)	67 (13.2)	
Other Enterobacteriaceae	49 (12.7)	18 (14.5)	67 (13.2)	
*Acinetobacter* spp	4 (1)	2 (1.6)	6 (1.2)	
*Pseudomonas* spp	18 (4.7)	14 (11.3)	32 (6.3)	
Other	3 (0.8)	2 (1.6)	5 (1)	
Source of bacteremia				.04
Urinary tract	269 (69.9)	83 (66.9)	352 (69.2)	
Primary, unknown source	20 (5.2)	16 (12.9)	36 (7.1)	
Abdominal/biliary	55 (14.3)	10 (8.1)	65 (12.8)	
Respiratory	14 (3.6)	6 (4.8)	20 (3.9)	
Central venous catheter	22 (5.7)	8 (6.5)	30 (5.9)	
Skin and soft tissue	5 (1.3)	1 (0.8)	6 (1.2)	
SOFA score at onset, median (IQR) [min, max]	2 (1–3) [0,11]	2 (1–3) [0,15]	2 (1–3) [0,15]	.05
Study arm- 7 days	193 (50.1)	62 (50)	255 (50.1)	.98
Vasopressor support	7 (1.8)	3 (2.4)	10 (2)	.67
Oxygen supplementation	34 (8.8)	18 (14.5)	52 (10.2)	.07
Infection management				
Appropriate empirical treatment administered within 48 h	339 (88.1)	97 (78.2)	436 (85.7)	.007
Physiotherapy consultation during hospitalization	55 (14.3)	43 (34.7)	98 (19.3)	< .001
Geriatrician consultation during hospitalization	9 (2.3)	9 (7.3)	18 (3.5)	.01
Hospital-acquired Pressure ulcer	10 (2.6)	11 (8.9)	21 (4.1)	.002
Severe diarrhea	21 (5.5)	13 (10.5)	34 (6.7)	.05
Length of hospital stay, median (IQR), days^d^	1 (0–3)	2 (0–6)	1 (0–3)	< .001
Discharge disposition				< .001
Home with family	280 (72.7)	85 (68.5)	365 (71.7)	
Home alone	63 (16.4)	12 (9.7)	75 (14.7)	
Nursing home	15 (3.9)	5 (4)	20 (3.9)	
Long term care facility	1 (0.3)	3 (2.4)	4 (0.8)	
Rehabilitation center	4 (1)	10 (8.1)	14 (2.8)	
Transferred to another hospital	0 (0)	1 (0.8)	1 (0.2)	
Other	22 (5.7)	8 (6.5)	30 (5.9)	
Hospital readmissions	128 (33.2)	63 (50.8)	191 (37.5)	< .001

^a^ Data are presented as number. (%) unless otherwise indicated.

^b^ Abbreviations: SOFA, sequential organ failure assessment.

^c^ Fourteen patients with bloodstream infection with Enterobacteriaceae had a polymicrobial infection (9 patients in the group of patients who experienced functional decline and in 5 patients who did not show functional decline). Of these 14 patients, 9 had another Enterobacteriaceae as a co-pathogen, 3 had Pseudomonas spp., and 2 had Aeromonas spp."

^d^ Length of hospital stay -per one day increment.

### Risk factors for functional decline

One out of four patients (124/509) showed functional decline at 90 days. In univariate analysis, patients who experienced functional decline tended to be older [76.5 years (IQR 64.3–83) vs. 69 years (IQR 58.5–78); *P <* .001], were dependent for IADL at baseline and were unemployed [93.4% (114/124) vs. 76.5% (286/385); *P <* .001] compared to those who did not show functional decline (Table 2). Other baseline characteristics associated with increased risk for functional decline were lower Norton score for risk of pressure ulcers (S1 Table [11]) and comorbidities including: congestive heart failure, cerebrovascular diseases, diabetes, chronic pulmonary disease, and malignancy. Infection characteristics associated with functional decline were higher Sequential Organ Failure Assessment (SOFA) score (S2 Table [12]) at infection onset and reduced consciousness at presentation. Hospital acquired bacteremia was also associated with increased risk of functional decline [38.7% (48/124) vs. 24.4% (94/385); *P =* .002]. Inclusion in the RCT’s intervention arm (7 days antibiotic treatment) was similar among patients who experienced functional decline and those who did not [50% (62/124) vs. 50.1% (193/385), *P =* .98, respectively. Table 3].

Patients who experienced functional decline were given less often appropriate empirical antibiotic treatment [78.2% (97/124) vs. 88.1% (339/385), *P =* .007]. During hospitalization patients who required oxygen supplementation, needed consultation of a physiotherapist or geriatric physician, experienced severe diarrhea or acquired pressure ulcers, were more prone to suffer from functional decline within 90 days. In addition, longer length of stay was associated with functional decline [7 days (IQR 5–12) vs. 5 days (IQR 4–7); *P <* .001, Table 3].

### Multivariable analysis

Multivariable analysis showed that older age (OR, 1.03; for an one-year increment, 95% CI 1–1.05), dependence for IADL at baseline (OR, 4.64; 95% CI 2.5–8.6), malignancy (OR, 2.01; 95% CI 1.14–3.55), chronic pulmonary disease (OR, 2.23 95% CI 1.12–4.42), low Norton score (OR, 0.87; 95% CI 0.79–0.96) longer hospital stay (OR 1.09; for an one-day increment, 95% CI 1.04–1.15) were independent risk factors for functional decline within 90 days. Appropriate empirical antibiotic treatment was associated with lower rates of functional decline within 90 days (OR, 0.4; 95% CI 0.21–0.78). SOFA score at infection onset and cerebrovascular disease were not independent risk factors for functional decline, Table 4.

**Table 4:** Multivariable analysis for factors associated with functional decline among 90 days survivors^a^.

Risk factor	Multivariable logistic regression analysis OR (95% CI)	Univariate analysis OR (95% CI)
Year of age ^b^	1.03 (1–1.05)*	1.04 (1.02–1.05)
Functional capacity at baseline		
Independent	Reference	Reference
Functional dependence in instrumental activities of daily living (IADL)	4.64 (2.5–8.6)*	6.74 (4.02–11.3)
Functional dependence in basic activities of daily living (BADL)	0.24 (0.1–0.6)*	1 (0.52–1.93)
Malignancy	2.01 (1.14–3.55)*	1.59 (1–2.51)
Cerebrovascular disease	1.99 (0.97–4.08)	1.96 (1.12–3.43)
Chronic pulmonary disease	2.23 (1.12–4.42)*	2.5 (1.44–4.32)
Norton score	0.87 (0.79–0.96)*	0.86 (0.8–0.92)
SOFA score at presentation	1.12 (0.98–1.28)	1.12 (1.01–1.25)
Hospital-acquired infection	1.65 (0.92–2.95)	1.96 (1.27–3.01)
Appropriate empirical treatment administered within 48 h	0.4 (0.21–0.78)*	0.49 (0.29–0.83)
Length of hospital stay	1.09 (1.04–1.15)*	1.12 (1.08–1.17)

^a^ Abbreviations: OR, odds ratio; CI, confidence interval.

^b^ Age-per one year increment; Length of hospital stay: per one day increment.

*Statistically significant *P <* .05.

Multivariable analysis using generalized linear models (GLM). N = 491, Akaike’s Information Criterion (AIC)– 428.936, constant β = -1.562.

## Discussion

In this observational study of 509 hospitalized patients with Gram-negative bacteremia, one out of four patients experienced functional decline within 90 days. Among patients who were functionally independent at baseline, 8% became dependent in basic ADL within 90 days of bacteremia onset. Furthermore, 13% of patients who were functionally dependent at baseline became bedridden within 90 days. Factors associated with increased risk for functional decline were: older age, dependency on others for IADL, malignancy, chronic pulmonary disease, low Norton score, inappropriate empirical treatment and higher length of hospital stay. High SOFA score at infection onset was associated with functional decline but did not emerge as such at multivariable analysis.

Dependent patients for IADL at baseline were at higher risk for functional decline within 90 days. Interestingly, patients with baseline dependence in basic ADL had low risk of functional decline. Similar results were demonstrated in a prospective study by Iwashyna et al. that showed sepsis patients with severe limitations at baseline did not experience significant functional decline compared to patients with normal functional capacity [8]. This finding was explained by a possible ceiling effect among this group of patients.

In our study, appropriate empiric antibiotic treatment was found to be a protective factor of functional decline. This outcome can be explained by a real effect or by a potential confounding factor that was not entered to our model. Studies investigating the impact of inappropriate empirical antibiotic therapy on short term mortality among patients with blood stream infections yielded conflicting results. While some studies have shown that inappropriate empirical antibiotic treatment increase mortality, [13–15] others have found no association [16, 17]. The controversy probably stems from heterogeneity in studies’ methodologies [13, 18, 19]. The impact of inappropriate empirical therapy on long term mortality was demonstrated in one study, showing significantly higher one-year mortality among patients with bacteremia receiving inappropriate empirical therapy [19]. However, the influence on functional capacity was not assessed.

We found that a large proportion of hospitalized patients with Gram-negative bacteremia experienced functional loss. Similar trends were observed in previously published studies. In a multicenter, prospective cohort study of 1,279 adults who were hospitalized for acute medical illnesses, 31% experienced functional decline between preadmission and hospital discharge. After three months, 40% of the cohort experienced disabilities in daily activities [20]. In concordance with our study results, a systematic review aimed to identify predictors of functional decline revealed that the most common risk factors are age, lower functional status, cognitive impairment, depression and length of hospital stay [21].

This study has several limitations. First, our study included only Gram-negative bacteremia patients who gave consent to participate in the RCT, this could limit the generalization of results. However, one of the RCT strengths was its nonrestrictive inclusion criteria that enabled a representative cohort of eligible patients [10]. Second, functional capacity at 30 and 90 days was assessed through telephone interviews. This could result in a self-reporting bias leading to either underestimation or overestimation of the outcome according to the patient’s subjective perception. Third, definitions and measurement methods of functional decline may differ between studies leading to difficulty in assessment of functional capacity. While many studies, including our RCT, examined all-cause mortality or other objectively assessed outcomes, this study assessed outcomes that impact patient’s quality of life; whether they will return to baseline activity level, what are the risk factors for functional decline and whether they can be prevented. In order to do this we only addressed patients who were alive on day 90.

## Conclusions

Functional decline is associated with an increased risk of mortality, and with higher health care costs [22, 23]. This study contributes to the identification of patients at risk of functional deterioration after hospitalization. We cannot expect patients with severe infections to return rapidly to their pre-infection functional status. At 90 days a quarter of patients surviving Gram-negative sepsis had a functional decline. These patients need support. Understanding the risk factors for functional decline is essential to clinicians, patients, their family members and health policy makers.

## Supporting information

S1 Table Norton score for risk of pressure ulcers. (DOCX)

S2 Table Sequential Organ Failure Assessment (SOFA) score. (DOCX)

S3 Table Charlson comorbidity index. (DOCX)

## Abbreviations and acronyms

RCT: Randomized Controlled Trial
I-ADL: Instrumental Activities of Daily Living
B-ADL: Basic Activities of Daily Living
IQR: Interquartile Range
OR: Odds Ratio
CI: Confidence Interval
SOFA: Sequential Organ Failure Assessment
