# Supplementary material for: Risk factors for functional decline among survivors of Gram-negative bloodstream infection: A prospective cohort study
Source: PLoS One. 2021 Nov 17;16(11):e0259707. doi: 10.1371/journal.pone.0259707 (PMC8598031; doi:10.1371/journal.pone.0259707)
Supplement: S1 Table — (DOCX) [file pone.0259707.s001.docx]

**S1 Table: Norton score for risk of pressure ulcers ^22^**

| **Score item** | **Answer choices (points)** |
| --- | --- |
| Physical condition | Good (4) Fair (3) Poor (2) Very bad (1) |
| Mental condition | Alert (4) Apathetic (3) Confused (2) Stupor (1) |
| Activity | Ambulant (4) Walk with help (3) Chair bound (2) Bed bound (1) |
| Mobility | Full (4) Slightly impaired (3) Very impaired (2) Immobile (1) |
| Incontinence | Not (4) Occasionally (3) Usually/Urine (2) Doubly (1) |
