# Supplementary material for: Risk factors for functional decline among survivors of Gram-negative bloodstream infection: A prospective cohort study
Source: PLoS One. 2021 Nov 17;16(11):e0259707. doi: 10.1371/journal.pone.0259707 (PMC8598031; doi:10.1371/journal.pone.0259707)
Supplement: S2 Table — (DOCX) [file pone.0259707.s002.docx]

**S2 Table: Sequential Organ Failure Assessment (SOFA) score ^23^**

| **SOFA score** | **0** | **1** | **2** | **3** | **4** |
| --- | --- | --- | --- | --- | --- |
| Respiration  PaO_2_/FiO_2_ (mmHg) | >400 | ≤400 | ≤300 | ≤200 | ≤100 |
| Coagulation  Platelets (*10^3^/mm^3^) | >150 | ≤150 | ≤100 | ≤50 | ≤20 |
| Liver  Bilirubin (mg/dL) | <1.2 | 1.2-1.9 | 2-5.9 | 6-11.9 | >12 |
| Cardiovascular  Hypotension ^a^ | No hypotension | MAP<  70 mmHg | Dopamine≤5  or dobutamine any dose | Dopamine>5  or adrenalin≤0.1  or noradrenalin≤0.1 | Dopamine >15  or adrenalin>0.1  or noradrenalin>0.1 |
| CNS  Glasgow Coma Score | 15 | 13-14 | 10-12 | 6-9 | <6 |
| Renal  Creatinine (mg/dL) | <1.2 | 1.2-1.9 | 2-3.4 | 3.5-4.9 or urine<500mL/d | >5 or urine<200mL/d |

^a^ adrenergic agents dosed at µg/kg/min
