# Supplementary material for: Risk factors for functional decline among survivors of Gram-negative bloodstream infection: A prospective cohort study
Source: PLoS One. 2021 Nov 17;16(11):e0259707. doi: 10.1371/journal.pone.0259707 (PMC8598031; doi:10.1371/journal.pone.0259707)
Supplement: S3 Table — (DOCX) [file pone.0259707.s003.docx]

**S3 Table: Charlson comorbidity index ^24^**

| **Score** | **Condition** |
| --- | --- |
| 1 | Coronary artery disease Congestive heart failure Chronic pulmonary disease Peptic ulcer disease Peripheral vascular disease Mild liver disease Cerebrovascular disease Connective tissues disease Diabetes Dementia |
| 2 | Hemiplegia Moderate-to-severe renal disease Diabetes with end-organ damage Any prior tumor (within 5 y of diagnosis) Leukemia Lymphoma |
| 3 | Moderate-to-severe liver disease |
| 6 | Metastatic solid tumor AIDS (not only HIV positive) |

Abbreviations: AIDS, acquired immune deficiency syndrome; HIV, human immunodeficiency virus.
